# Mobilization of mercury from lean tissues during simulated migratory fasting in a model songbird

**DOI:** 10.1038/srep25762

**Published:** 2016-05-12

**Authors:** Chad L. Seewagen, Daniel A. Cristol, Alexander R. Gerson

**Affiliations:** 1 Natural Resources Department, AKRF Inc., White Plains 10601, NY, USA; 2 Great Hollow Nature Preserve, New Fairfield 06812, CT, USA; 3 Biology Department, College of William & Mary, Williamsburg, VA 23187, USA; 4 Department of Biology, University of Massachusetts, Amherst, MA 01003, USA.

## Abstract

The pollutant methylmercury accumulates within lean tissues of birds and other animals. Migrating birds catabolize substantial amounts of lean tissue during flight which may mobilize methylmercury and increase circulating levels of this neurotoxin. As a model for a migrating songbird, we fasted zebra finches (*Taeniopygia guttata*) that had been dosed with 0.0, 0.1, and 0.6 parts per million (ppm) dietary methylmercury and measured changes in blood total mercury concentrations (THg) in relation to reductions in lean mass. Birds lost 6–16% of their lean mass during the fast, and THg increased an average of 12% and 11% in the 0.1 and 0.6 ppm treatments, respectively. Trace amounts of THg in the 0.0 ppm control group also increased as a result of fasting, but remained extremely low. THg increased 0.4 ppm for each gram of lean mass catabolized in the higher dose birds. Our findings indicate that methylmercury is mobilized from lean tissues during protein catabolism and results in acute increases in circulating concentrations. This is a previously undocumented potential threat to wild migratory birds, which may experience greater surges in circulating methylmercury than demonstrated here as a result of their greater reductions in lean mass.

Methylmercury is a toxic and bioavailable form of mercury that can be prevalent in aquatic and terrestrial food webs^1^. Unlike other lipophilic contaminants, which accumulate in fatty tissues (e.g., organochlorine pesticides), methylmercury accumulates within lean tissues because of its affinity for the sulfhydryl groups of proteins^2^,^3^. It easily crosses the membranes of lean tissue cells due to its high lipid solubility and covalently bonds with thiols within the cytoplasm^4^. The liver, kidneys, and muscles are the tissues in which birds and other vertebrates typically accumulate the most mercury^2^,^5^.

In migrating birds, organs and muscles are rapidly catabolized during flight, losing as much as 50% of their pre-flight mass^6^, as a way to maintain water balance, lower transportation costs, and provide citric acid cycle intermediates that are needed to sustain prolonged fatty acid oxidation^7^,^8^. The magnitude of reduction in each tissue appears to be linked to its protein turnover rate, as does the degree of methylmercury accumulation^5^,^6^. The organs and muscles that undergo the greatest mass reductions during flight are therefore often those that have the highest methylmercury concentrations. Similar to the mobilization of lipid-bound contaminants that can occur when animals draw on their fat stores^9^,^10^, we have previously proposed that methylmercury that has accumulated in the organs and muscles of migrating birds throughout the preceding wintering or breeding season could be rapidly released into the bloodstream during in-flight protein catabolism^11^,^12^. This could result in acutely high concentrations of circulating methylmercury and possibly affect neurological functions that are important to migration performance and survival^11^.

As a model for the lean mass dynamics that occur in migrating songbirds^6^,^13^,^14^, we subjected methylmercury-dosed zebra finches (*Taeniopygia guttata*) to a prolonged fast and measured changes in their blood mercury concentrations in relation to changes in their lean mass. We expected fasting to increase blood mercury level and that the increase would be proportional to the loss of lean tissue.

## Results

Dosing resulted in average blood total mercury concentrations (all forms of mercury; THg) of 0.987 (±0.213 SD) and 5.749 (±1.092 SD) μg/g (hereafter ppm) in the 0.1 and 0.6 ppm groups, respectively. Control birds had only traces of blood THg (mean: 0.006 ppm ± 0.003 SD; range: 0.003 to 0.011 ppm). Males and females did not differ in blood THg concentration, lean body mass, or the amount of lean body mass lost during the fast (Wilk’s λ = 0.97, *F*_3,40_ = 0.36, *p* = 0.78).

Starting lean mass and change in lean mass were not different among treatment groups (Wilk’s λ = 0.91, *F*_4,80_ = 0.94, *p* = 0.45). Birds lost 0.626–2.385 g (mean: 1.545 g ± 0.409 SD) of lean mass during the fast, which represented 6–16% of their starting lean mass (mean: 12% ± 3 SD).

**Figure 1:**
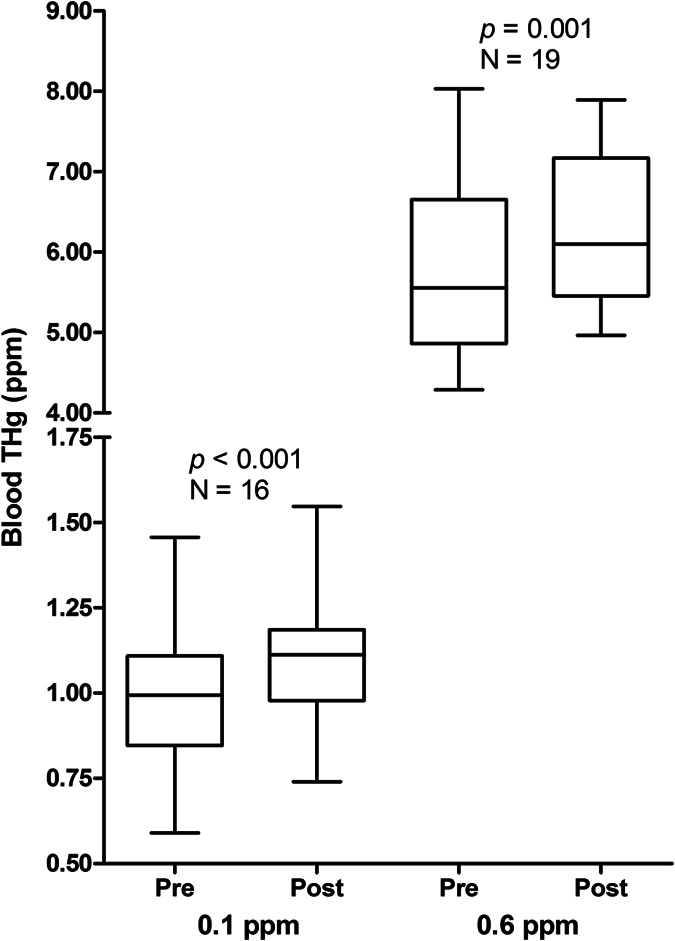
Pre- and post-fasting blood total mercury (THg) concentrations in zebra finches dosed with 0.1 or 0.6 ppm methylmercury-cysteine. Boxes show the median and 25^th^ and 75^th^ percentiles; whiskers show minimum and maximum values. Probability values are from paired t-tests of pre- and post-fasting blood THg concentrations of birds within each dosing group.

Fasting significantly increased blood THg in the 0.1 ppm (*t*_15_ = 5.40, *p* < 0.001) and 0.6 ppm treatments (*t*_18_ = 6.27, *p* = 0.001) by an average of 12% and 11% above starting concentrations, respectively (Fig. 1). The trace amounts of blood THg in the control group also increased after fasting (*t*_8_ = 3.00, *p* = 0.017), but concentrations remained extremely low (mean = 0.009 ± 0.005 ppm).

**Figure 2:**
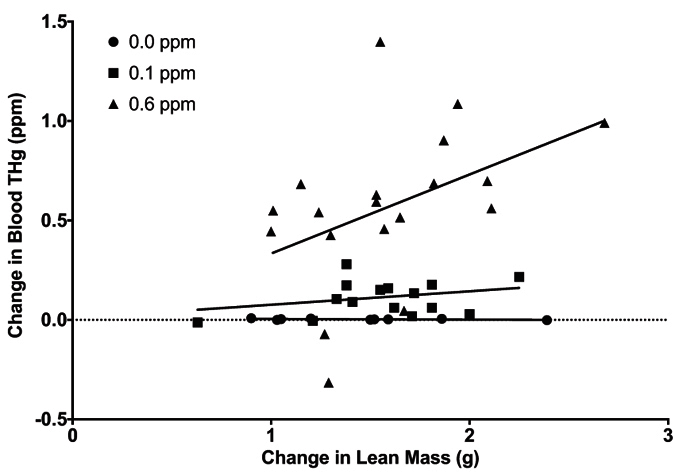
Changes in blood total mercury (THg) concentration in relation to lean mass loss in zebra finches dosed with 0.0, 0.1, or 0.6 ppm methylmercury-cysteine.

Overall, change in blood THg was largely explained by change in lean mass (*F*_1,40_ = 3.74, *p* = 0.06).When we tested treatments separately, there was a marginally significant relationship between change in blood THg and change in lean mass for the 0.6 ppm group (*F*_1,17_ = 1.95, *p* = 0.07), but not for the 0.1 ppm (*F*_1,14_ = 1.33, *p* = 0.26) or 0.0 ppm groups (*F*_1,7_ = 2.25, *p* = 0.18; Fig. 2). The slope of the model for the 0.6 ppm group indicated that blood THg increased an average of 0.40 ± 0.20 ppm for every 1 g of lean mass catabolized.

## Discussion

Blood THg concentrations increased by over 10% during fasting, indicating that mercury was mobilized from lean tissues as birds catabolized endogenous protein to satisfy their metabolic requirements. The increases were unlikely to have been caused by reductions in blood volume because birds are notably able to maintain plasma and red blood cell volume under dehydrating conditions^15^,^16^, and the amount of blood collected was negligible. We found the increases in blood THg to be largely explained by reductions in lean mass in the high dose group, documenting for the first time the extent to which mercury in the lean tissues of birds can be mobilized during fasting, and presumably long-distance migratory flight.

No relationship was detected in the 0.1 ppm group, which suggests a dose-dependent effect of protein catabolism on blood THg that is likely driven by the amount of mercury that has accumulated in the tissues. We predict that longer exposure as well as higher dietary concentrations would result in a more pronounced relationship between lean mass loss and increase in circulating THg.

The relationship between change in blood THg and change in lean mass was also likely affected by individual variation in organ and muscle mercury concentrations among birds within the same treatment. Conspecific birds on a common dosing regime exhibit high variation in organ and muscle mercury levels due to differences in demethylation and elimination abilities among individuals^17^. In the wild, we suspect this greatly influences the degree to which in-flight protein catabolism increases circulating mercury levels, possibly providing a means by which migratory bird populations undergo selection for individuals that more readily demethylate and eliminate mercury. The ability of female birds to transfer dietary mercury into their eggs may have contributed additional individual variation in organ and muscle mercury levels among zebra finches within the same treatment group and further weakened our ability to detect a relationship between change in blood THg and change in lean mass at the lower dosing level.

It has been shown that high protein turnover tissues, such as liver and small intestine, are catabolized more so during flight and fasting than slower protein turnover tissues, such as skeletal muscle^6^. There may be a switchover period where protein is first catabolized from tissues that are relatively low in mercury, such as the gizzard and intestines^18^,^19^, or carbon is derived from the small free amino-acid pool instead of from lean tissue. Later in the fast, tissues in which the most mercury accumulates (e.g., liver, kidneys) may become the primary sources of catabolized protein. The source and relative amount of protein contributed to the fuel mixture during endurance flight can also depend on the size of fat stores^20^. All of this would yield variable patterns of mercury mobilization depending on the progression of the animal through the different stages of fasting.

Our birds lost an average of only 12% of their starting lean mass during the fast, which is well below the 30–50% reductions that commonly occur in migrating birds^21^,^22^. From the corresponding average increases in blood THg of 11–12% that we observed in the two dosing groups, we can speculate about potential increases in blood THg in wild migratory birds that undergo more substantial reductions in lean mass. Assuming a comparable pool of mercury available for mobilization from lean tissues, a *Catharus* thrush, for example, that departs its wintering or breeding grounds with a blood THg level of 0.3 ppm^23^ and catabolizes 40% of its lean mass^24^ might experience an increase of around 40% (0.12 ppm) during the first leg of its migration. Circulating mercury is readily available to cross the blood-brain barrier [2–3; cf. 25], raising questions about how these surges in blood mercury might affect the neurological processes associated with orientation, flight, and stopover refueling behaviors^11^,^12^. Also important to determine are the kinetics of mercury mobilized during migratory fasting in order to better understand the magnitude and duration of circulating concentrations and the pattern of redeposition during refueling. Our study represents an important step towards determining whether the ubiquitous and increasing global pollutant mercury, like some other contaminants^26^, might affect the vitality of migrating birds during this vulnerable phase of their life cycle.

## Methods

### Study species and dosing

Although they are non-migratory, the pattern of protein degradation that occurs in zebra finches fasted at rest is largely the same as that which occurs during in-flight fasting in migrating birds, with the liver, kidneys, intestines, flight muscles, and gizzard showing the most pronounced reductions in mass^6^,^13^,^14^. Zebra finches are therefore a useful model for the study of lean tissue catabolism that is representative of migrating birds^13^.

We fed captive-bred, adult zebra finches Zupreem Fruit Blend® diets dosed with either 0.0, 0.1, or 0.6 ppm methylmercury-cysteine for approximately 8 wk. Birds on the same diet were housed together in 3 × 2.5 × 2 m outdoor aviaries with *ad libitum* food and water. Food was prepared as described previously^27^, and each batch was tested and rejected if it was >7.5% different from the nominal concentration. The 0.0 ppm treatment represented a control group to demonstrate that mercury did not influence the degree of lean mass loss during fasting, and the dosing levels of 0.1 and 0.6 ppm were intended to reflect a low and high range of mercury concentrations found in invertebrate prey of songbirds in the eastern U.S.^28^,^29^,^30^. Sample sizes were 9 (7 males, 2 females), 16 (10 males, 6 females), and 19 (10 males, 9 females) for the 0.0, 0.1, and 0.6 ppm treatment groups, respectively.

### Body composition

We measured pre- and post-fasting fat mass and lean mass using quantitative magnetic resonance (QMR) analysis (Echo Medical Systems, Houston, USA) as in^8^,^24^,^31^. QMR analysis provides fast, highly accurate, non-invasive measurements of fat and lean mass in small vertebrates, including zebra finches, to the nearest 0.001 g^31^,^32^. Birds were scanned in duplicate on the “two accumulation” setting of the EchoMRI software and the duplicate measurements were then averaged (all CV <3%)^8^,^24^. The machine was calibrated according to manufacturer instructions and a 17 g canola oil standard was scanned periodically to confirm accuracy.

### Fasting

Postabsorptive birds were weighed, blood sampled (~100 μL by brachial venipuncture), scanned in the QMR, and then held in indoor cages without food or water for 18–21 h. Following the fast, birds were weighed, scanned in the QMR, and blood sampled from the opposite wing. All procedures were approved and overseen by, and carried out in accordance with the regulations of, the College of William & Mary Institutional Animal Care and Use Committee.

### Mercury analysis

We measured THg (ppm wet weight) in the blood, which is representative of methylmercury in birds^23^,^33^, using cold vapor atomic absorption spectroscopy as described by^34^. Mean percent recoveries of standard reference materials were 95.1% (DOLT-4) and 99.0% (BCR463-tuna). The detection limit of the mercury analyzer was checked regularly and averaged 0.005 ng, which was below the quantities of THg detected in the blood of the non-dosed control birds.

### Statistical analyses

We used multivariate analysis of variance (MANOVA) to assess whether sex had any effect on pre-fasting blood THg concentrations, pre-fasting lean mass, or the amount of lean mass lost during the fast because sex ratios were different among treatment groups. Pre-fasting lean mass and change in lean mass after fasting were compared among treatment groups also using MANOVA. Blood THg before and after the fast was compared within each treatment group using paired t-tests. We used a general linear model (GLM) to test the relationship between change in blood THg and change in lean mass. The initial model included treatment group, change in lean mass, change in fat mass, and all two-way interactions as factors. Non-significant terms were then sequentially dropped (all *p* > 0.17) and the final model included treatment group and change in lean mass. We also tested the relationship between change in blood THg and change in lean mass within each treatment group independently using GLMs. Change in lean mass, change in fat mass, and change in blood THg data were log + 1 transformed prior to analyses to better meet assumptions of homoscadicity and normality. Tests were performed using R and statistical significance was accepted when *p* < 0.05.

## Additional Information

**How to cite this article**: Seewagen, C. L. *et al.* Mobilization of mercury from lean tissues during simulated migratory fasting in a model songbird. *Sci. Rep.* **6**, 25762; doi: 10.1038/srep25762 (2016).
